# Data Analysis Planning and Reporting for Confirmatory Multi‐Lab Preclinical Trials: A Tutorial

**DOI:** 10.1002/bimj.70152

**Published:** 2026-07-27

**Authors:** María Arroyo‐Araujo, Clarissa F. D. Carneiro, Sophie K. Piper, Juliane C. Wilcke, Nicole Ellenbach, Anne‐Laure Boulesteix, Robbert Emprechtinger, Benjamin V. Ineichen, Lars Bjorn Riecken, Bernhard Haller, Bernhard Voelkl, Leonhard Held, Frank Konietschke, Ulf Toelch

**Affiliations:** ^1^ Berlin Institute of Health Charité ‐ Universitätsmedizin Berlin QUEST Center for Responsible Research Berlin Germany; ^2^ Institute of Biometry and Clinical Epidemiology Charité – Universitätsmedizin Berlin Berlin Germany; ^3^ Institute of Medical Informatics Charité – Universitätsmedizin Berlin Berlin Germany; ^4^ Institute for Medical Information Processing, Biometry, and Epidemiology LMU Medizin LMU Munich Munich Germany; ^5^ Department of Clinical Research University of Bern Bern Switzerland; ^6^ Leibniz Institute on Aging Fritz Lipmann Institute Jena Germany; ^7^ Institute of AI and Informatics in Medicine TUM School of Medicine and Health Technical University of Munich Munich Germany; ^8^ Animal Welfare Division University of Bern Bern Switzerland; ^9^ Epidemiology Biostatistics and Prevention Institute (EBPI) and Center for Reproducible Science (CRS) University of Zurich Zurich Switzerland

## Abstract

Confirmatory multi‐lab preclinical trials are a powerful experimental strategy to enable decisions to transition from preclinical to clinical settings. With their complexity, such study designs pose several challenges in statistical planning, analysis, and reporting of experiments. To address these, we convened an expert group of biostatisticians and biomedical scientists currently involved in such trials to summarize in a tutorial the most common challenges and offer general guidance. Furthermore, we incorporated statistical advice from existing clinical trials’ guidelines and adapted it into recommendations for preclinical trials. We describe strategies on key topics such as calculating sample sizes, handling differences between centers, and selecting relevant covariates. Additionally, we give guidance on statistical methods to account for lab effects and proper reporting of analyses. We embed this in a general discussion on remaining open questions to advance the analysis of preclinical confirmatory studies. The provided general, non‐case‐specific guidance serves as a conversation starter between biomedical scientists and statisticians to develop robust statistical analysis strategies for confirmatory multi‐lab preclinical trials.

## 1 Introduction

Preclinical efficacy studies, that is, studies using animal or cell models to assess the efficacy of a treatment, often inform decisions to start clinical trials. Recently, however, low rates of translation of new treatments to humans (Ineichen et al. 2024) and low rates of replicability among preclinical studies prompted researchers to reconsider how preclinical studies ideally inform clinical research (Dirnagl 2019; Errington et al. 2021; Leenaars et al. 2019).

One important reason for low translation rates and reproducibility is the limited generalizability (i.e., external validity) of findings across experimental settings that vary with respect to biological and environmental factors (Voelkl et al. 2021). To probe boundary conditions and increase generalizability, systematic variations (heterogenization) are introduced in multi‐laboratory (multi‐lab) preclinical trials (Carneiro et al. 2023; Hunniford et al. 2023). That is, each laboratory follows harmonized and standardized protocols to collect experimental data, and between‐lab variation is attributed to known and unknown differences between labs (Hunniford et al. 2023). For example, between‐lab variation affects outcome variability to a greater extent than varying the mice‐breeding site (Jaric et al. 2024).

With their increased rigor, multi‐lab studies fill the important role of confirmation in translation from preclinical to clinical settings (Hunniford et al. 2023; Kimmelman et al. 2014). Confirmations here extend replications. Replications play an important role in probing the results of an exploratory study by repeating the study as closely as possible. Confirmatory multi‐lab study protocols are systematically adjusted and potentially vary not only from exploratory study protocols but also between laboratories. Some of these changes may be due to logistics, necessary reagent changes, or the introduction of a second sex to experiments. For this reason, confirmation meaningfully extends replications by acknowledging, mapping, and systematically introducing differences to exploratory studies, making such studies distinct from replications in the narrow sense. Such studies will have increased credibility of the research findings and thus have high value in evaluating the underlying knowledge claims (Dirnagl 2019; Huang et al. 2020; Kimmelman et al. 2014; Mogil and Macleod 2017; Nosek et al. 2025). Therefore, confirmatory multi‐lab preclinical trials have been proposed as an essential part of improving translation (Chamuleau et al. 2018; Maertens et al. 2017). Whereas funders have established calls specifically for these studies (Bundesministerium für Bildung und Forschung 2018, 2022), it is currently unclear how the novel structure of these trials would be best reflected in the statistical analysis plan (SAP).

To give guidance, we convened a workshop with biostatistical, clinical, and preclinical researchers to review and discuss best practices for confirmatory multi‐lab preclinical trials. Furthermore, we reviewed official guidelines for the analysis of clinical trials to inform the preclinical debate (Biostatistics | European Medicines Agency (EMA) 2014). Similarly to clinical trials, there is no one‐size‐fits‐all strategy. Rather, we present a tutorial including the most common challenges and key steps for the analysis of preclinical multi‐lab trials regarding planning, implementing, and transparently reporting the methods relevant to the presented results. This will support an initial dialogue between biomedical scientists and statisticians to develop and refine robust statistical analysis strategies for confirmatory multi‐lab preclinical trials.

## 2 The Study Design Forms the Basis of Analysis

**FIGURE 1:**
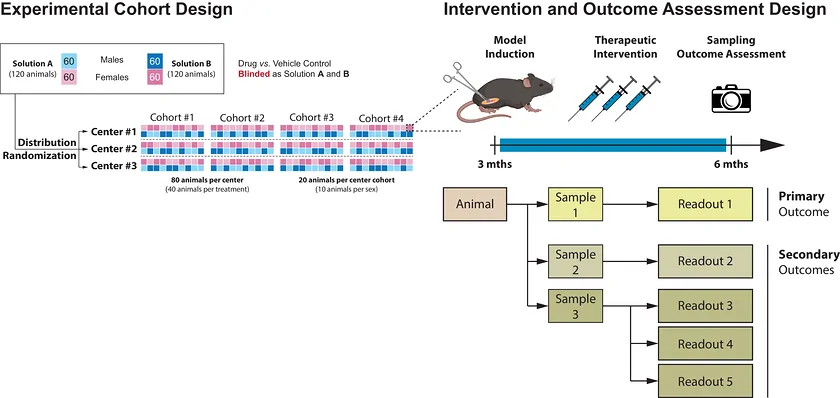
Example experimental design chart. On the left side, the distribution of animals is shown for each batch/cohort, lab/center, treatment, and sex. On the right side, the timeline of experimental procedures is shown, and the outcome measures are listed with a clear distinction between experimental units (animals) and subunits (samples from the same animal). An applied example is available in Figure S1. mths: months.

The analysis of confirmatory multi‐lab preclinical trials begins during the planning stage of the project. An experimental design chart is a useful tool to recap the main study features of interest, such as labs, treatment groups, and other variables relevant to carry over to the analysis (Figure 1). Experimental design in multi‐lab preclinical trials has been discussed previously (Drude et al. 2022); once available, it should be the scaffold of the SAP. Although there is limited guidance on what should be addressed in an SAP for preclinical studies (Aban and George 2015), randomized controlled clinical trials offer guidance that can be partly adapted to the preclinical scenario (see Gamble et al. 2017; Piper et al. 2023; Stevens et al. 2023).

Briefly, the SAP describes the prespecified analytical framework, including outcome definitions, sample size considerations, data acquisition and preprocessing, variable construction and transformation, specification of statistical models, decision criteria, adjustment for multiple comparisons, planned alternative and sensitivity analyses, and strategies for addressing missing data and other anticipated analytical challenges.

Importantly, the SAP needs to a priori address the key decision‐making points at different stages of the trial. This concerns decisions such as when to stop the trial early and under which circumstances, what to do if a treatment‐by‐lab interaction is found but no (interpretable) main effect of the treatment (see Section 4.4), and how a successful confirmation will be declared. While defining the key decision‐making points, it is crucial to keep the purpose of the trial firmly in mind. Preclinical multi‐lab trials commonly generate evidence about a treatment effect to inform decisions on whether to move on to clinical research (Drude et al. 2022). When this is the case, the decision‐making points specify which outcome(s) and expected result will inform the decision of moving on to clinical testing, or further testing the hypothesis due to inconclusive results. For preclinical studies, it is highly recommended to budget for a statistician for the study team. The qualified statistical advice is necessary throughout the whole project to prevent unintended data mishandling.

If feasible, the analysis code is part of the predefined SAP. Preliminary data, such as pilot or simulated data, used to identify and support statistical decisions are disclosed as such. With some masking adaptations, the analysis code or pipeline executes the confirmatory analyses blindly (i.e., without knowing the treatment allocation) to prevent the introduction of biases. In addition, it is recommended to keep records of when the blinding was broken and the analysis was finalized. An SAP is prespecified in several different formats, from writing it on a paper/electronic lab notebook to a highly recommended digital open preregistration (see Box 1 for further details).

**Box 1: Confirmation requires (pre‐)registration**

Preregistration of a study and its SAP offer several key benefits, including preventing unintentional analytical biases and enhancing transparency, credibility, and reproducibility. By submitting a study and SAP to a repository before data collection, researchers make the analyses and decisions traceable a priori (Nosek et al. 2018), preventing p‐hacking and HARKing: “Hypothesizing After the Results are Known” (Kerr 1998). Preregistration also allows all labs involved in a trial to follow a consistent approach in data collection and reporting, and it makes exploratory analysis clearly distinguishable from confirmatory ones. Additionally, the preregistration is confidential (i.e., under embargo) until publication, addressing intellectual property concerns. Various repositories, such as OSF (https://osf.io/), PreclinicalTrials (https://preclinicaltrials.eu/), and Animal Study Registry (https://www.animalstudyregistry.org/), are available for preclinical studies.

Moreover, a registered report embeds preregistration directly in the publication process. Here, a study is submitted to a journal, peer‐reviewed, and eventually accepted for publication prior to data collection. This process is based only on its methods and SAP, reducing publication biases (Chambers and Tzavella 2022).

In summary, preregistration strengthens the credibility of confirmatory research by ensuring that analysis strategies are prespecified and transparently shared.

Regarding content, SAPs require sufficient information for other researchers to be able to perform the analyses independently (Kahan et al. 2020). Through this, researchers will be able to move faster from the end of data collection to the communication of their findings. A concrete checklist for the content of SAPs based on formal guidelines exists for clinical trials (Gamble et al. 2017), and it can be adopted for preclinical trials with modifications based on the topics described in this article on a case‐by‐case basis.

Note that deviations from the planned analyses are possible at any stage, as long as they are clearly and transparently justified and reported (see Section 5). In addition, once data are collected, review is conducted without any group/treatment identifiers to minimize the risk of introducing biases (EMA 1998).

Tightly linked to the SAP is the DMP (data management plan). Herein, research teams specify handling and storage of raw and processed data with proper documentation. Developed before the start of the experiment, it considers details such as the volume and formats of data to obtain, how long data should be stored, and who is allowed to reuse it, resulting in a metadata description of collected data. Nowadays, funders require a DMP for submitting an application. For more details on the DMP, see (Pergl et al. 2019; Michener 2015).

## 3 Current Common Approaches to Multi‐Lab Analysis

The prevailing current practice in preclinical research is to adapt methods from single‐laboratory studies to multi‐lab studies, potentially resulting in a suboptimal analysis and misleading interpretation of results. A systematic assessment of in vivo multi‐lab preclinical studies exhibited a broad variety of analytical strategies (Hunniford et al. 2023). Among their sample of studies, 11 analyzed data from each lab independently (Bramlett et al. 2016; Browning et al. 2016; Dixon et al. 2016; Gill et al. 2015; Jha et al. 2021; Jones et al. 2015; Kliewer et al. 2020; Llovera et al. 2015; Mountney et al. 2016; Reimer et al. 1985; Shear et al. 2016), eight pooled the data from all labs (Alam et al. 2009; Arroyo‐Araujo et al. 2019; Crabbe et al. 1999; Gill et al. 2015; Jones et al. 2015; Llovera et al. 2015; Reimer et al. 1985; Spoerke et al. 2009) and only two of these considered the lab as variable in the analytical model (Arroyo‐Araujo et al. 2019; Crabbe et al. 1999). In addition, two studies combined the multi‐lab data in a meta‐analysis (Llovera et al. 2015; Maysami et al. 2016). Note that four studies presented multiple analyses of the same dataset (Gill et al. 2015; Jones et al. 2015; Llovera et al. 2015; Reimer et al. 1985) and that there was also considerable variation in study designs (Hunniford et al. 2023). This diversity of strategies highlights a lack of consensus in the field, partly due to missing guidelines.

During our workshop, two analytical methods were identified as the most common approaches currently employed in (preclinical and clinical) multi‐lab studies; both methods are based on linear models adapted to various experimental designs, namely regression models and analysis of variance (ANOVA). Regression models are a robust approach to coping with data missing at random and unbalanced designs. Missing data will, however, require imputation methods like multivariate imputation by chained equations (MICE) to adequately address this (Azur et al. 2011; Brunner et al. 2018). As in clinical trials, data that is not missing at random will require an advanced investigation.

The analytical flexibility in choosing which variables to include and how to include them (i.e., fixed or random effects) can introduce bias, but this risk can be reduced by preregistration of the SAP. When regression model assumptions are violated, nonparametric rank‐based alternatives are available; they offer less flexibility than parametric methods such as mixed models and are less straightforward to interpret. ANOVA is a special case of a linear model where handling missing data for longitudinal studies (i.e., repeated‐measures ANOVA) is complex and sometimes not possible. Moreover, it is not suitable for quantitative covariates, which may require extension to analyses of covariance. Similar to regression models, if parametric assumptions are violated, nonparametric (rank‐based) methods are the less flexible alternative.

Additionally, methods specialized for clinical designs are used for analysis with complex study designs. For example, a recent large‐scale multi‐lab preclinical trial in stroke adopted the multi‐arm multi‐stage adaptive design (Jennison and Turnbull 1999; Neumann et al. 2017). In the aforementioned trial, six different interventions were tested for their efficacy across six laboratories in four stages. Through interim futility tests, inefficacious interventions were removed, and only interventions that exceeded a futility threshold proceeded to the next testing stage (Lyden et al. 2022). Planning and conducting such trials are highly resource‐intensive, though, and require an experienced statistician to plan and perform the analyses.

## 4 Key Items to Include in the SAP of Preclinical Confirmatory Multi‐Lab Trials

There are different approaches to develop the SAP for a preclinical confirmatory multi‐lab study. As a starting point, we considered the EMA guidelines for the analysis of multicenter trials in clinical studies (EMA 1998). Further guidelines describe the use of covariates in the analysis (EMA 2015), particularly the consideration of center effects, the use of sensitivity analyses to improve robustness (EMA 2020), the adjustment of baseline variables (EMA 2015), and handling of missing data (EMA 2010). These topics are discussed in the context of preclinical multi‐lab confirmatory trials below.

Although this section highlights the items considered most relevant for confirmatory multi‐lab trial analysis, it is not an exhaustive review of all possible scenarios that researchers might encounter while writing the SAP for this type of experiment. Therefore, in sections where we found it necessary, we included a “reality check” outlining potential solutions in case the initial SAP proved suboptimal after data acquisition. Given that the suggested solutions in the ‘reality‐check’ sections address scenarios after data acquisition, these alternatives have to be added to the SAP as a posteriori amendments and reported as such in related publication(s) (see Lakens 2024 for guidance on how to best present deviations from pre/specified protocols). Failing to report these amendments transparently potentially misleads readers and limits the value of and trust in the results and their interpretation.

### 4.1 Selecting the Outcome Variables of Interest

First, outcome variables collected in the trial need to be clearly defined, including the exact description of any transformations or calculations that were applied to the raw data. For example, if the outcome is a score composed of several measures (e.g., several behavioral tests to compose a neurobehavioral score) or if the raw data are obtained as a measure with arbitrary units (e.g., optical density) and a normalized outcome is more biologically meaningful, then the formula to obtain the main outcome of interest should be explicitly stated.

Furthermore, within the confirmatory framework, primary and secondary outcomes of interest need to be distinguished. This, in turn, is relevant for informing confirmatory and exploratory results accordingly, which will then guide the next steps in the research pathway (Kimmelman et al. 2014).

The primary outcome will indicate whether the confirmation was successful and signal progress toward clinical application. It will further inform the study sample size and statistical power calculations. Ideally, it reflects a clinically relevant outcome. Although this is not available for all preclinical models and core preclinical outcome sets have not been widely developed, the COMET Initiative database includes clinical core outcome sets that can be consulted and adapted for preclinical trials (COMET Initiative | Core Outcome Measures in Effectiveness Trials n.d.).

Similarly, secondary outcomes will provide supporting evidence on the main knowledge claim sought, based on secondary objectives or triangulation of evidence from multiple preclinical models and/or methods (e.g., animal models combined with ‐omics analysis). Secondary outcomes provide, for example, causal or mechanistic insights into the research question that inform new trials. If the trial is not powered for the secondary outcomes, caution must be taken when interpreting test statistics, and results must be treated as exploratory findings.

### 4.2 Determining a Study Sample Size

Next, it is necessary to establish the reliability of the trial. A sample size calculation ensures that the study achieves the required statistical power. If the sample size is restricted by ethical, practical, or financial reasons, the minimum effect size will indicate the effect size that the experiment is able to detect under an a priori fixed power and fixed sample size. That is, the sample size is fixed along with the power level to estimate the effect size (Krzywinski and Altman 2013). This alternative, however, is only applicable in rare situations (e.g., animal models with high severity), and priority should be given to selecting an informed sample size according to, for example, the smallest effect size of interest (Danziger et al. 2022). It has been previously suggested that exploratory effect sizes tend to be inflated (Colquhoun 2014; Danziger et al. 2022), this overestimation is often referred to as “winner's curse” and a consequence is the “regression to the mean” (van Zwet and Cator 2021); therefore, the inflation of the exploratory effect size should be considered when using it for the sample size calculation in follow‐up experiments (Colquhoun 2014); otherwise, follow‐up studies risk being underpowered and thus less informative.

Additionally, the sample size calculation rests on the relevant experimental unit. The experimental unit signifies the unit that is independently and randomly assigned to the treatment conditions (Hurlbert 1984; Lazic et al. 2018), such as a mouse or a vial with frozen cells. The experimental unit is often distinct from the observational unit, which is the outcome generated by the experimental unit, for example, behavioral or histological readings from a mouse, multiple well‐plate readings coming from the same vial of cells. Anticipated missing data, due to, for example, possible failures in model induction or during intervention or other modes of attrition, increase sample sizes further.

Finally, the sample size calculation needs to correspond to the envisioned statistical analyses as closely as possible. For example, it accounts for any pairing or stratification in the data and specific assumptions of the statistical test/model used in the primary analysis. In complicated designs, simulations inform sample size calculation (Brazma et al. 2001; Wilson et al. 2021). Typically, small sample sizes in preclinical studies make it challenging to stratify for all factors, and a subset needs to be selected. If violations of test assumptions occur, more appropriate methods should be chosen (e.g., nonparametric, resampling, or Bayesian; Box 2). Note, however, that such alternatives will potentially not test the same hypotheses in the same way, thus possibly changing the results interpretation. A general summary of helpful multi‐lab EMA (EMA 2006).

Importantly, the multi‐factorial structure of the statistical model is an important factor during the sample size calculation, in particular, laboratory effects and possible interactions. In multi‐lab studies, observations from the same lab are more similar than those from different labs (i.e., animals/samples have been housed, handled, and assessed in the same way). That is, data are not truly independent of the lab and, thus, laboratory effects must be taken into account (Vierron and Giraudeau 2007). Although there is no specific guidance on how to incorporate this in the planning of preclinical trials, one recommendation is to perform a single sample size calculation for the multi‐lab trial and perform stratified randomization on laboratories, similarly to multicenter clinical trials (Vierron and Giraudeau 2007).

Regarding possible interactions, it depends on the type of interaction that is expected, whether the power and the sample size have to be modified. If detecting an interaction is a priority for the study, it is necessary to specify the relevant interaction effect size and then calculate the sample size correspondingly (Carneiro et al. 2023). It is worth noting that powering studies for interactions results in drastic increases in the sample size.

**Box 2: Bayesian approaches as an alternative to address common problems**

Currently, preclinical data analysis mainly relies on frequentist statistical methods, such as null hypothesis significance testing, which are often criticized for their limited value for decision‐making (Amrhein et al. 2019; Harrell 2017; Hoekstra et al. 2018; Stahel 2021). In contrast, the Bayesian framework has several advantages, particularly for preclinical research. One key advantage is the inclusion of external information (known as priors), which makes Bayesian approaches more flexible and interpretable. For example, a meta‐analytic predictive approach can reduce the required sample size by estimating the prior effective sample size to reduce the number of experimental units needed or to adapt the randomization ratio, making experiments more feasible and cost‐effective. (Bonapersona et al. 2021; Schmidli et al. 2014; Unseld 2023). See Giovagnoli 2021 for an overview of the Bayesian adaptive design.

Bayesian methods are particularly useful for repeated testing, as they can mitigate the inflation of false positives by focusing on whether a treatment meets a certain threshold rather than relying on a null hypothesis. Additionally, it allows researchers to directly assess the increase in confidence/credibility achieved by the confirmatory study, supporting evidence‐based decisions on whether to proceed to clinical research.

Finally, Bayesian approaches could be extended to decision‐making frameworks that incorporate multiple outcomes, helping to identify, for instance, the best cost‐effectiveness based on the best efficacy and least adverse events from a set of alternative actions (Berry et al. 2010; Ryan et al. 2020).

### 4.3 The Role of Control Groups and Baseline Measures

Another fundamental aspect to address in an SAP for a confirmatory multi‐lab trial is the role of control measurements (Dehue 2000). Two elements are important: (1) experimental controls and (2) baseline measures. Experimental controls are conditions designed to isolate the causal effects of a specific treatment or intervention, acting as benchmarks for drawing valid and reliable conclusions. There are two types of experimental controls: technical controls ensure the consistency of the data obtained, whereas biological controls help to understand the biological variability among experimental units.

In contrast, baseline measures encompass characteristics collected from animals or samples before undergoing any intervention (Vickers and Altman 2001). In clinical research, differences in the characteristics of the samples or patients are referred to as baseline differences, as researchers have limited control over patients who enroll. In multi‐lab preclinical trials, differences in population characteristics are small due to genetically homogeneous animals. Moreover, no issues in enrolling animals in the study are expected, as the sample of animals tested tends to come from a common origin and the same protocol will be applied across all laboratories. Nevertheless, differences in unaccounted population characteristics can emerge due to failed homogenization across laboratories or gene–environment interactions (Voelkl and Würbel 2021), but can be mitigated through systematically heterogenized designs (Carneiro et al. 2023; Voelkl et al. 2021). Differences also emerge due to deficits in the experimental design and suboptimal research practices (e.g., insufficient bias reduction) or attrition bias (i.e., different rates of loss or exclusion of animals per experimental group). With that, differences in animal/sample characteristics that are strongly linked to outcomes potentially bias intervention effect estimates and need to be explored and reported (Collazo et al. 2023; Hewitt et al. 2010; Welch et al. 2023).

Whereas tests of baseline sample characteristics imbalance have no value in truly randomized trials (Senn 1998), multi‐lab preclinical trials should report unintended differences in baseline characteristics/measurements across groups and labs as described above. Some differences can be prevented through unified protocol development and personnel training, but they are unavoidable, for example, in the case of equipment differences. To address this, performing manipulation checks in the study design to ensure animals react as expected to experimental manipulations (Hoewe 2017).

As a general recommendation, the SAP of a confirmatory trial includes a priori definitions of progression or stopping criteria as often as possible. For example, what if there are differences in baseline measurements between labs that would lead to the exclusion of data from individual labs? Importantly, the exclusion of data points or labs needs to be transparently reported in the experimental design chart, similar to clinical trials. Here, differences between technical controls across labs also contribute to an evaluation of the outcome assessment method used in the experiment. For further recommendations on how to best address baseline measures and control groups, see Piper et al. (2023).

Through the adoption of open data policies, baseline reference datasets for comparison become available more frequently and potentially inform the analysis plans of confirmatory trials (Abbasi 2023). These previous studies provide data for the use of historical controls or inform the selection of covariates regardless of baseline differences. These are contentious points even in clinical trials (van Rosmalen et al. 2018); however, more research is needed to assess the value of using reference datasets in preclinical studies.

**Reality check: control groups and baseline measurements**

When baseline differences are unavoidable, one option is to transform the outcome measurement to a relative effect size (e.g., normalizing by experimental controls), though this potentially masks the variability between labs. An assessment of the analytical strategy on a case‐by‐case basis will again benefit from a statistician's input.

### 4.4 Inclusion of Covariates and Their Interactions

Ideally, randomization of experimental units to experimental groups is organized centrally (EMA 1998). In the SAP, details of the randomization factors are included alongside used methods. Important prognostic or diversifying variables (e.g., disease severity, age, sex, batch) are recommended to be taken as stratification factors in the randomization procedure, which will in turn be included as potential covariates in the statistical model.

The selection of covariates to include in the analysis, that is, any categorical or continuous variable, needs to be carefully discussed while planning the study, and it should identify the experimental factors that are expected to influence the main outcome variable (EMA 1998). When new insights on potentially relevant covariates emerge from the data in exploratory analyses, these need to be clearly labeled as such and not be confused with confirmatory analyses. In line with this, it is not recommended to adjust the main analysis for covariates that were measured after the experiment took place, as these could be affected by the treatment/intervention (EMA 1998).

Three important points need to be considered for the selection of covariates in confirmatory multi‐lab preclinical trials: the number of covariates, whether interactions can be expected, and the choice between using the covariates as fixed or random factors.

Ideally, all relevant covariates should be included when assessing the efficacy of an intervention. There is, however, the possibility that models will become overparameterized when too many variables are included. That is, underlying data are not sufficient to reliably estimate all effects of interest, particularly in studies with small sample sizes. Therefore, it is best to identify in advance the most important covariates and justify their relevance for the research question (e.g., biological or clinical relevance).

Interactions of variables with the treatment effect require particular attention. Treatment effects potentially rely on population characteristics such as strain, sex, or age. If sample size calculations only consider the main effect of interest (e.g., treatment effect across groups/doses), the study is underpowered for detecting most interaction effects (Carneiro et al. 2023). In the presence of an interaction, main effect interpretation is challenging, particularly when the interaction is partially attenuated (i.e., where the effect size of one group is higher than the other, but both follow the same direction of the effect thus do not lower the power to detect the main effect; Carneiro et al. 2023), resulting in *p*‐values that are not diagnostic with regard to the null hypothesis. Multi‐lab studies often result in even more complex interactions if there are interactions between more than two variables, for example, treatment‐by‐lab and sex or age. Such interactions between three or more covariates will be challenging to detect and to interpret; thus, it is important to be aware of and transparent about these. Given the complexity of the interpretation of such interactions, we discourage testing for interactions of order higher than two; this will keep the analysis interpretable.

The covariates selected will cluster observations within the values this covariate can take (e.g., within each sex, within each cage, within each age class). Independent variables can be modeled as either fixed or random effects; one caveat of this is that exact definitions of these effects vary (Gelman 2005). Random effects account for variability among a large group of units that are random representatives of a larger group. Hence, this model assumes that group‐level effects stem from a distribution of effect sizes that reflects heterogeneity between groups. Underlying assumptions and subsequent choice of effect type will be particularly important to discuss a priori for each outcome of interest within a multi‐lab preclinical trial.

**Reality check: covariates and interactions**

In case the model is overparameterized, one solution is a dimension reduction (e.g., principal component analysis (PCA) or autoencoder; Wang et al. 2016). This, however, potentially obscures meaningful covariates that reflect features of the population and/or experimental conditions. In addition, results interpretation is challenging as modeled covariates contain multiple related variables (Dyer and Kording 2023). Thus, this approach is best used in a priori defined analyses (e.g., preregistration of SAP) (Dyer and Kording 2023; Shinn 2023).

Alternatively, model assessment methods like information criteria akaike information criterion (AIC), bayesian information criterion (BIC) identify more parsimonious models (see Akaike 1998 for details), whereas least absolute shrinkage and selection operator (LASSO) methods shrink the regression coefficients to key variables (Tibshirani 1996). These methods require careful implementation and statistical advice. Ultimately, the goal is to balance model fit and complexity to obtain interpretable and parsimonious models.

### 4.5 Laboratory as a Dedicated Covariate

Whereas we cannot discuss all possible experimental variables, the location of data collection is specific to multi‐lab trials. The variability (controlled or uncontrolled) between labs can limit statistical conclusions, but it improves external validity in confirmatory research (Voelkl et al. 2018; Voelkl and Würbel 2021).

The conduct of experiments between laboratories should be kept as standardized as possible. That is, all labs apply the same protocol. Selected factors that vary between labs are documented and investigated for a potential influence on the outcome (Arroyo‐Araujo et al. 2022; Carneiro et al. 2023). The adjustment for lab effects is recommended if there are expected differences between labs, even despite the homogenization of protocols, or if the lab was used as a stratification factor during randomization. Not adjusting could lead to incorrect standard error estimates for the treatment effect (Kahan and Harhay 2015). If not included in the analysis plan, this particular decision has to be made before unmasking the group identities of the data points (EMA 2020).

In terms of the number of observations per lab, the standard recommendation for clinical multicenter studies is to keep the variation of observations per center as low as possible and to avoid having too small centers, as this too would lead to the recommendation of not including centers in the statistical model (EMA 1998). In preclinical trials, however, there is greater control over how many observations are expected per lab. Therefore, sample size calculations reflect the structure of the experimental design as described in Section 4.2.

Additionally, to the number of observations per lab, it is necessary to consider the number of laboratories involved. Whereas guidelines of clinical trials do not define how many centers are expected in small or large multicenter efforts, it is safe to assume that preclinical trials will typically not reach what is considered “large” in clinical settings.

To give more concrete recommendations on how the number of laboratories influences the accuracy of results, simulations have been conducted. Based on published preclinical data, they indicate that experiments performed in four labs had the most accurate effect size compared to single‐lab studies without requiring a bigger sample size (Voelkl et al. 2018). In line with this, statistical adjustment for preclinical multi‐lab analysis to account for the interaction of “phenotype‐by‐laboratory” (or gene‐by‐laboratory, GxL; Voelkl et al. 2020) showed increased replicability compared to single‐lab experiments at the cost of a small power reduction (Jaljuli et al. 2023). Moreover, the fact that they used three labs to test the replicability of the results suggests three as an appropriate number of labs to include in a multi‐lab preclinical study. This balances the strength of evidence with the resources needed to implement a multi‐lab study. Nevertheless, it has been discussed that even two labs already improve reproducibility (Voelkl et al. 2018) when laboratories are entered as a fixed covariate. If, in the case of only two labs, one lab fails to complete experiments or produces vastly different results, the multi‐lab character is lost, and data interpretation will be challenging.

The choice between treating the lab as a fixed or random factor in analyses is complex. Arguments against treating labs as a random effect include the potential for inaccurate variance estimates due to the small number of labs involved, minimal benefits of using a mixed model with only two labs, and technical difficulties in parameter estimation due to small sample sizes in preclinical studies (e.g., convergence). Further, there is the potential for unintentional bias in confirmatory studies through increased flexibility of mixed effects models, as random effects structures need to be specified. Arguments in favor include the conceptual soundness of this approach, research suggesting comparable performance of mixed models even with few levels (Gelman and Hill 2006), and the better distinction between the variable of interest and the nuisance variable. The final decision will depend on a careful consideration of these factors within the specific context of the study. To summarize, multi‐lab preclinical trials should consider the lab effect and its possible interactions as fixed instead of random factors (EMA 1998) unless otherwise justified, and include the GxL interaction whenever possible.

Once the number of laboratories and observations per lab has been settled, it is necessary to consider how this factor will be handled in relation to other factors in the analysis, given its central role in the trial. According to the ICH E9 guideline (EMA 1998) for clinical trials, the main treatment effect should be tested across centers without a center‐by‐treatment interaction term, as it might reduce the efficiency of the test for the main effects. If the treatment effect is heterogeneous across labs, interpretation of the main treatment effect needs to consider causes for heterogeneity, as this would potentially suggest that further studies are needed before the treatment effects can be estimated reliably. It is unclear whether the same will apply to preclinical trials, particularly considering the low number of labs. Furthermore, as also discussed in Section 4.2, multi‐lab trials are not expected to be adequately powered for interaction effects, so any conclusion from these analyses is exploratory (Agresti and Hartzel 2000; EMA 1998; Senn 1998).

## 5 Reporting the Results

Equally important to planning and performing research is the dissemination of a trial. Here, it is helpful to consult guidelines that promote clear and comprehensive reporting of methods and results. Until there are reporting guidelines for confirmatory preclinical studies available, check whether reporting guidelines specifically for the topic of the preclinical study (e.g., Fisher et al. 2009; Shineman et al. 2011) or for the specific data types used (e.g., Brazma et al. 2001; Tipton et al. 2014) exist already (e.g., through literature search or on https://www.equator‐network.org). Otherwise, more general clinical guidelines exist for adaptation (e.g., SPIRIT, Chan et al. 2013; CONSORT, Schulz et al. 2010). To give an example, the frequently used ARRIVE 2.0 guidelines (Percie du Sert et al. 2020) for reporting animal research are easily expanded by the relevant points mentioned in Drude et al. (2022) for reporting confirmatory trials. Reporting of attrition in animal experiments is transparently reported by following the four phases mentioned in the CONSORT statement (Schulz et al. 2010), namely enrollment, allocation, follow‐up, and analysis being relevant for preclinical trials.

Regarding the confirmatory aspect of the study, it is important to avoid selective reporting of the results, in particular HARKing (Kerr 1998) or different forms of p‐hacking (Stefan and Schönbrodt 2023). This means that all hypotheses and planned analyses from the analysis plan are reported, even if the results are unexpected or unsatisfactory (e.g., nonsignificant results). Additional results like sensitivity analyses, subsample analyses, triangulation, and other exploratory analyses will facilitate a holistic understanding of the study. For this, it must also be disclosed which results, analyses, and visualizations are confirmatory according to the analysis plan and which parts are exploratory, whether planned or unplanned. If there have been unexpected but reasonable changes or deviations from the analysis plan, these must be reported transparently and fully, for example, by first describing each change, then giving a rationale, and judging its effect on the study results (cf. transparent changes template of the OSF Preregistration Challenge at https://osf.io/yrvcg, updates in Animal study registry and version control in preclinical trials).

For full transparency of the statistical analyses and obtained results as well as their reproducibility, it is necessary to state all software and packages, including the version numbers used for the analyses of the data, for example, full details in the electronic supplement or included with the open data. It is also possible to assign a resource research identifier (RRID) to R code: RRID:SCR_003005. Deposit all raw data and metadata (i.e., information on the data to make them usable by others) and all scripts for the analyses and visualizations in a data repository adhering to findable, accesible, interoperable, re‐usable (FAIR) principles together with the DMP (Wilkinson et al. 2016). Avoid “data available upon request” statements as it is proven an inefficient strategy to ensure data sharing (Gabelica et al. 2022; Tedersoo et al. 2021). Tables and an in‐text description of comprehensive summary results (e.g., means, standard deviations, confidence intervals, test conducted, test statistics, exact *p*‐values) will further facilitate the reuse of data and results.

Besides transparency in reporting, clear and informative visualizations are also extremely relevant for unbiased interpretation of the findings. In particular, regardless of whether or how the analysis takes the lab into consideration, visualizing the variability within and between labs can provide insights into potential interactions or differences in baseline measurements (Weissgerber et al. 2019).

**FIGURE 2:**
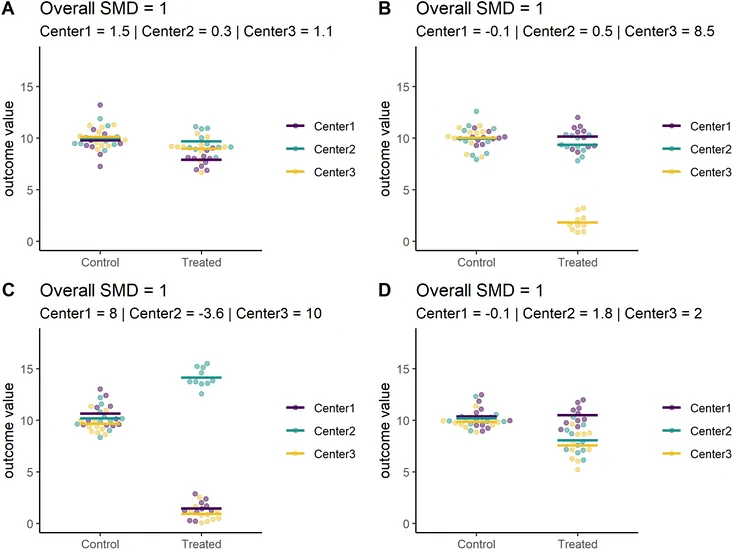
Visualizing variability within and between labs. Simulated datasets are shown where the overall effect size is the same across four different scenarios: (A) all labs find effects in the same direction; (B) two labs find nearly no effect and one lab finds a large effect; (C) two labs find effects in one direction and one lab finds an effect in the opposite direction; (D) one lab finds no effect at all and two labs find consistent effect sizes. In all plots, each experimental unit is presented as a small translucent circle, and the mean of each group is presented for each lab as a horizontal line.

There are several possibilities for visualizing multifactorial designs. Here, we highlight one solution to visualize variability (Lord et al. 2020), adapting it so that it presents the variability within and between labs. In Figure 2, we display four examples of how the labs’ effects can vary while the treatment has the same effect size when data from all labs is pooled. The same visualization strategy is applicable if exploring all factors included in the statistical model (e.g., sex, cage, batch), so that there is a clear representation of how variability is distributed across each factor level.

## 6 Discussion

Preclinical multi‐lab confirmatory trials have been suggested to meaningfully add to evidence‐based decision making as they evaluate a research claim rather than the replicability of a result (Chamuleau et al. 2018; Kimmelman et al. 2014; Maertens et al. 2017). Strengths include an efficient use of resources (e.g., animal numbers) for a given preclinical trial due to an increase in reproducibility of methods and credibility of results, given the meticulous implementation of robust research practices (Hunniford et al. 2023; Voelkl et al. 2018). As preclinical multi‐lab confirmatory trials become more frequent, additional challenges will arise (Drude et al. 2022). Designing, planning, and conducting these studies require extensive resources, including time. Here, we highlight key challenges associated with the statistical analysis and reporting of such studies. Through expert elicitation, we seek to raise awareness about specific considerations that apply in multi‐lab settings and encourage preclinical researchers and statisticians to collaboratively plan rigorous and decision‐critical experiments.

Although we are unable to provide a step‐by‐step analysis protocol or thorough guidance of planning and analysis of confirmatory multi‐lab preclinical trials, we hope to provide a general roadmap of topics to be addressed in an analysis plan. Importantly, the statistical analysis starts at the offset of the study planning, and it is strongly recommended that statisticians are consulted as early as possible. The sample size calculation represents a crucial procedure through which the experimental plan is mapped on the statistical plan, so it needs to be scientifically informed and statistically guided for the resulting data to be robust and meaningful.

Three main topics are of high priority to be discussed between scientists and statisticians to include in the pre‐defined SAP: control and baseline measures, selection of covariates, and how to consider the multi‐lab aspect of the design. A priori definition of criteria for considering baseline differences or differences between control groups across labs as acceptable or not will greatly improve the trustworthiness of the analysis results. If such differences were unexpected, solutions can involve data transformations, looking for open datasets to establish a historical database, or deeper investigations of the labs’ effects.

Regarding covariates, the main recommendation is to select as many as needed for the biological interpretation of the experimental findings and as few as possible to avoid over‐parameterization. In many situations, including covariates as fixed‐effects factors is a statistically valid and straightforward solution. However, this consideration will still need to be carefully discussed in each individual study. Beyond this, interactions between covariates can provide additional insights about the main effect of interest, but if the study was not powered for interactions, these results are exploratory and need to be transparently reported as such.

The factor lab needs to be included in the statistical model and, consequently, also in the sample size planning in preclinical trials by including lab as a stratification factor in the randomization. In case the number of laboratories is small, the lab is then included as a fixed‐effects factor in the statistical model. For large studies, both in number of labs and sample size, the lab is modeled as a random factor (Chu et al. 2011; Clark and Linzer 2015; Dieleman and Templin 2014; Kahan 2014; Kahan and Morris 2013; Pickering and Weatherall 2007). For multi‐lab preclinical studies, treatment‐by‐lab interaction is not a priority, but the aim of having multiple labs is to increase the generalizability of the study across different contexts.

Statisticians provide valuable support in the interpretation of results from a mathematical model, but biomedical scientists need to be knowledgeable enough about the statistical findings to properly link them to the biological interpretation of the results. For example, assumptions about the hypothetical underlying rate of effective interventions for a given field should have a major impact on how a *p*‐value is interpreted (Held 2010; Ioannidis 2005; Krzywinski and Altman 2013; Nuzzo 2014). Understanding the strengths and limitations of different statistical frameworks or approaches is also key from the planning of the study, through interpreting and reporting of results, to making decisions regarding the translational potential of the findings (Hoekstra et al. 2018; Wasserstein and Lazar 2016).

A limitation to the perspectives presented here is that we have gathered a group of preclinical researchers and statisticians working on projects funded under the same call (Bundesministerium für Bildung und Forschung 2018) or who are specially interested in preclinical research and reproducibility. As preclinical multi‐lab trials become more common and are recommended more often, formal guidelines at national or international levels will be valuable for the field. We have incorporated as many relevant factors as feasible from clinical guidelines, as there is extensive literature available on the analysis and statistical planning of clinical trials. However, the goals of clinical trials and preclinical efficacy trials differ. Clinical trials obtain evidence on efficacy and safety to obtain regulatory approval and are necessarily heavily regulated. Preclinical trials rather test the possibility that these drugs will be potentially efficacious in humans. They should effectively inform decisions to start clinical trials and reduce the risk of later translational failures. On top of these, there are methodological differences like budget availability, variability of the data, number of labs, and cases within labs included in multi‐center/lab trials, among others. Therefore, not all recommendations for clinical trials are thus applicable to preclinical trials. Here, we incorporated recommendations by EMA for clinical trials, but we want to emphasize that preclinical trial designs and analyses need to take these different goals into account. Moreover, the recommendations outlined in this article aim to an ideal scenario, whereas in reality some practices and analytical choices are less feasible than others, in which case we strongly recommend to clearly document the decisions made and their justification and to transparently report them in the article and/or project repository.

Additional research is needed to determine the best practice to meet the goals of preclinical research. In particular, under what circumstances do such multi‐lab trials provide evidence that is decision‐enabling? How can the increased effort for such trials be justified, and do such trials actually help weed out interventions that later prove to be inefficacious? Nevertheless, this tutorial serves as a useful resource for bridging gaps in the collaborations between biomedical scientists and statisticians and as a solid discussion starter in supporting the development of proper analysis strategies for preclinical multi‐lab confirmatory trials.

## Author Contributions

**M.A.‐A.:** Conceptualization, Project administration, Writing – original draft, and Writing – review & editing. **C.F.D.C.:** Conceptualization, Project administration, Visualization, Writing – original draft, and Writing – review & editing. **S.K.P.:** Writing – review & editing. **J.C.W.:** Writing – original draft and Writing – review & editing. **N.E.:** Methodology, Writing – original draft, and Writing – review & editing. **A.‐L.B.:** Writing – review & editing. **R.E.:** Writing – original draft. **B.V.I.:** Resources, Writing – original draft, and Writing – review & editing. **L.B.R.:** Visualization and Writing – review & editing. **B.H.:** Writing – review & editing. **B.V.:** Writing – review & editing. **L.H.:** Writing – review & editing. **F.K.:** Writing – review & editing. **U.T.:** Conceptualization, Funding acquisition, Supervision, Writing – original draft, and Writing – review & editing.

## Conflicts of Interest

The authors declare no conflicts of interest.

## Code Availability Statement

A description of the simulation and the code used to generate Figure 2 is given in the Supporting Information files and is available at https://doi.org/10.5281/zenodo.13746419.

## Supporting information



Example of an experimental design chart of a sciatic nerve tumor model.**Supporting File 1**: bimj70152‐sup‐0001‐SuppMat.png.

**Supporting File 2**: bimj70152‐sup‐0002‐SuppMat.zip.

## Data Availability

The data that support the findings of this study are available in the Supporting Information of this article.
