# Supplementary figures and images for: Data Analysis Planning and Reporting for Confirmatory Multi‐Lab Preclinical Trials: A Tutorial
Source: Biom J. 2026 Jul 27;68(4):e70152. doi: 10.1002/bimj.70152 (PMC13402973; doi:10.1002/bimj.70152)

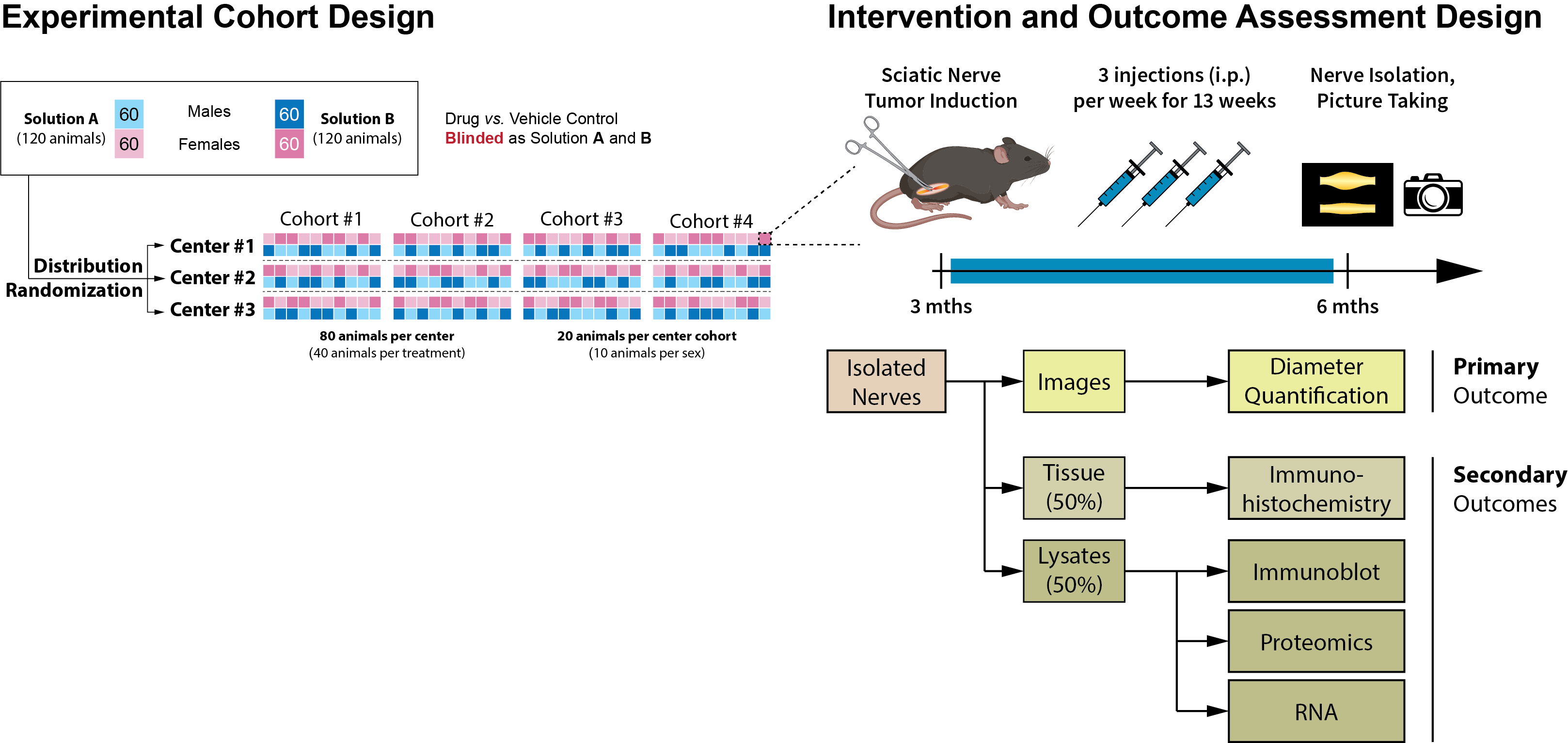

Supplement: Supplementary file 1 — Example of an experimental design chart of a sciatic nerve tumor model.Supporting File 1: bimj70152‐sup‐0001‐SuppMat.png. [file BIMJ-68-e70152-s001.png]
